# Systemically Administered Ligands of Toll-Like Receptor 2, -4, and -9 Induce Distinct Inflammatory Responses in the Murine Lung

**DOI:** 10.1155/2011/746532

**Published:** 2011-03-22

**Authors:** H. Ehrentraut, R. Meyer, M. Schwederski, S. Ehrentraut, M. Velten, C. Grohé, P. Knuefermann, G. Baumgarten, O. Boehm

**Affiliations:** ^1^Department of Anesthesiology and Intensive Care Medicine, University Hospital Bonn, Sigmund-Freud-Straße 25, 53105 Bonn, Germany; ^2^Institute of Physiology II, University of Bonn, Nussallee 11, 53115 Bonn, Germany; ^3^Department for Pneumology, Evangelische Lungenklinik, Berlin, Lindenberger Weg 27, Haus 205, 13125 Berlin, Germany

## Abstract

*Objective*. To determine whether systemically administered TLR ligands differentially modulate pulmonary inflammation. *Methods*. Equipotent doses of LPS (20 mg/kg), CpG-ODN (1668-thioat 1 nmol/g), or LTA (15 mg/kg) were determined via TNF activity assay. C57BL/6 mice were challenged intraperitoneally. Pulmonary NF*κ*B activation (2 h) and gene expression/activity of key inflammatory mediators (4 h) were monitored. *Results*. All TLR ligands induced NF*κ*B. LPS increased the expression of TLR2, 6, and the cytokines IL-1*αβ*, TNF-*α*, IL-6, and IL-12p35/p40, CpG-ODN raised TLR6, TNF-*α*, and IL12p40. LTA had no effect. Additionally, LPS increased the chemokines MIP-1*α*/*β*, MIP-2, TCA-3, eotaxin, and IP-10, while CpG-ODN and LTA did not. Myeloperoxidase activity was highest after LPS stimulation. MMP1, 3, 8, and 9 were upregulated by LPS, MMP2, 8 by CpG-ODN and MMP2 and 9 by LTA. TIMPs were induced only by LPS. MMP-2/-9 induction correlated with their zymographic activities. *Conclusion*. Pulmonary susceptibility to systemic inflammation was highest after LPS, intermediate after CpG-ODN, and lowest after LTA challenge.

## 1. Introduction

Severe sepsis is still the leading cause of death in surgical intensive care units and can be held responsible for approximately 9% of deaths per year in USA and Germany [1]. In the course of ongoing sepsis, many organs develop dysfunction. In this context, the lung can either be the source of infection or is remotely affected by systemic inflammation as bacterial degradation products reach this organ via the bloodstream. In both cases, an extensive release of proinflammatory cytokines and chemokines induces migration of neutrophils, fibroproliferation, and reorganization of lung tissue [2]. An accompanying disruption of the alveolar-capillary interface and consecutive leakage of protein-rich fluid into the interstitial and alveolar space causes hypoxemia and reduces lung compliance. Acute lung injury (ALI) or its severe form, the acute respiratory distress syndrome (ARDS), constitute clinical manifestations of the pathological process and develop in 40% of the patients suffering from sepsis [3]. Deaths from ALI or ARDS contribute significantly to mortality and morbidity [4].

Toll-like receptors (TLRs) act as pivotal signalling proteins of the inflammatory response during sepsis. They are activated by bacterial, viral, and fungal products. Gram-negative bacteria release the TLR4 ligand lipopolysaccharide (LPS) [5] whereas the cell wall component lipoteichoic acid (LTA) from gram-positive bacteria is primarily bound by TLR2 [6]. Both, TLR2 and TLR4 are localized in the cell membrane while TLR9 is localized in lysosomes and recognizes bacterial DNA from both gram-positive as well as gram-negative bacteria. Bacterial DNA contains CpG-ODN motifs [7], which are unmethylated CG dinucleotides prevalent in bacterial but not in mammalian DNA [8]. TLRs are differentially expressed in many organs including heart and lung as well as various cell types [9].

It has been shown that TLRs are involved in a number of inflammatory diseases of the lung, such as allergic asthma [10], autoimmune lung injury [11], and pneumonia [10]. Bacterial components induce the expression of inflammatory mediators such as proinflammatory cytokines and chemokines, which are highly relevant for the pathogenesis of pulmonary inflammation and injury [12–14]. Both attract and activate immune cells infiltrating the lung. Also, during sepsis, they participate in disrupting the alveolo-capillary structure, rupture of basal membranes, and interstitial matrix remodelling. The integrity of the extracellular matrix (ECM) is controlled by a dynamic equilibrium of synthesis and local degradation. Matrix metalloproteinases (MMPs) are the main physiological mediators of ECM degradation, which are also upregulated under pathological conditions like pulmonary inflammation or sepsis. They are primarily regulated by a class of endogenous inhibitors, tissue inhibitors of MMPs (TIMPs).

There has been a significant gain of knowledge about the high complexity of organ-specific TLR signalling in the lung. This organ exhibits a TLR pattern, which derives in part from parenchymatous cells as well as from immune cells like neutrophils. Hence, it may be speculated that the inflammatory response depends on specific TLR stimulation, and thus, various virulence factors might induce different inflammatory responses. It can be hypothesized that different organs express specific TLR patterns, which serve their need in defense against pathogens. An accurate characterization of TLR expression and signalling as well as the investigation of consecutively induced inflammatory mediators [15] is essential for understanding a differential inflammatory response to varying stimuli. It might be the fundament for developing efficient strategies for diagnosis and treatment of sepsis. However, the number of studies comparing the influence of different TLRs *in vivo* is rare [16]. In particular, a comparison of ligands for TLR2, -4, and -9 *in vivo* in the lung has not yet been performed. Therefore, we analysed the influence of three different TLR ligands (LTA, LPS, and CpG-ODN) on the expression of TLRs, cytokines, chemokines, MMPs, and TIMPs in the murine lung. Also, the subsequent activities of MPO, MMP-2, and -9 and the level of TNF-*α* protein in pulmonary tissue were investigated. The stimuli were applied remotely (intraperitoneal, i.p.) to simulate the progression of sepsis. 

## 2. Material and Methods

### 2.1. LPS, LTA, and CpG-ODN Preparations

Lipopolysaccharide (LPS) was from Escherichia coli (E. coli, 0 : 111, Sigma Aldrich Chemie GmbH, Munich, Germany). Lipoteichoic acid (LTA) was kindly provided by S. Morath, University of Konstanz, Germany (charge MGM5-10) and prepared as described before [17]. The LPS contamination of the LTA preparations was less than 1 EU/mg as determined by the LIMULUS amoebocyte lysate assay (Charles River, Charleston, SC). An immunostimulatory CpG-ODN (1668 thioat, sequence: GCTAGACGTTAGCGT) was purchased from Tib-MolBiol (Tib-MolBiol GmbH, Berlin, Germany).

### 2.2. Experimental Animals

158 male C57BL/6 mice 12 weeks of age from Charles River (Sulzfeld, Germany) were incorporated in the study. Mice were housed in pathogen-free cages with free access to water and standard rodent chow. Mice (*n* = 3–6/group) were treated with i.p. injections of PBS or different TLR ligands, that is, equipotent doses (see below) of either LTA (15 mg/kg), LPS (20 mg/kg), or 1668 thioat (1 nmol/g). PBS administration served as control. 30 min before stimulation with CpG-ODN, mice received 1 mg/kg D-Galactosamine (D-GalN; Roth, Karlsruhe, Germany; in control experiments D-GalN alone did not induce an inflammatory response; data not shown). At the end of the experiments, mice were sacrificed under anaesthesia with isoflurane 2.5 Vol.% (Forene, Abbott GmbH, Wiesbaden, Germany). The animals were handled according to the principles of laboratory animal care (NIH publication no. 85-23, revised 1996), and animal procedures were approved by the local committee for animal care.

### 2.3. TNF Activity Assay

C57BL/6 mice (*n* = 3/group) were stimulated with LPS (20 mg/kg i.p., according to [18]), CpG-ODN (1 nmol/g i.p., according to [19]), or LTA (15 mg/kg i.p., according to [20]). To determine potency of the applied concentrations of the three TLR ligands, undiluted serum from stimulated mice was tested *in vitro* on fibroblast cultures. The test of serum was chosen, as during a remote inflammation virulence factors are transported to the lung via the blood stream. We used a TNF activity assay according to a protocol published before [21]. Briefly, 2 h after stimulation, serum was taken, and murine fibroblast tumor cells were incubated with this serum and stained to determine viability. Binding of TNF-*α* and -*β* to surface receptors initiates lysis in certain types of cells. The TNF activity assay employs TNF-sensitive, actinomycin D-treated murine L929 fibroblasts to quantify TNF activity. Murine fibroblast tumor cells were grown in RPMI 1640 medium containing 10% fetal calf serum (FCS), 5 mM L-Glutamin, 25 mM HEPES, 5 mM sodium pyruvate, and 100 U penicillin and streptomycin, respectively. A 96-well plate containing 5 × 10^4^ cells per well was incubated over night in a humidified incubator (37°C, 93% O_2_, 7% CO_2_). Medium was removed and fresh serum or medium or TNF-*α* standard (rTNF, Sigma Aldrich, Munich, Germany) were added. 10 *μ*L of 1 : 50 diluted actinomycin D (Sigma Aldrich) was given to each well and incubated over night. After removal of the supernatant, cells were fixed with 5% formalin for 10 min. Then, wells were washed with PBS. 100 *μ*L of 0.05% Crystal Violet (Sigma Aldrich) in 20% ethanol was added to each well followed by destaining with tab water, and, finally, plates were dried. Absorbance was measured at 590 nm after dilution of the stain with 100 *μ*L methanol per well. Increased staining and absorbance corresponded to increased L929 fibroblast viability and decreased lytic effects of TNF. 

### 2.4. Pulmonary Nuclear and Cytoplasmic Extraction

Pulmonary protein extracts were prepared using the NE-PER Nuclear and Cytoplasmic Extraction Reagents (Pierce Biotechnology, Rockford, IL, USA) according to the manufacture's protocol and as published previously [22]. Briefly, lung tissue was pulverized on dry ice. The tissue weight was determined, and the appropriate amount of CER I (10-fold excess over the weight of tissue) containing 0.5 mg/mL benzamidine (Roche, Basel, Switzerland), 2 *μ*g/mL aprotinin (Roche), 2 *μ*g/mL leupeptin (Roche, Basel, Switzerland), and 0.75 mM PMSF (Roche, Basel, Switzerland) was added. The homogenates were vortexed and incubated for 10 min on ice. CER II was added, incubated again for 1 min on ice, and centrifuged for 5 min at 13,200 U/min (16,110 × g) and 4°C. The supernatant was transferred to a prechilled tube and used as cytoplasmic fraction for analysis of zymographic activity. The pellet was resuspended in ice-cold NER. After vortexing and incubation according to manufacturer's protocol, the sample was centrifuged again for 10 min at 16,110 × g. The supernatant was immediately transferred to prechilled tubes and used as nuclear extract for determination of NF*κ*B-DNA binding activity. The protein concentration was assessed by use of a bicinchoninic acid assay kit (Pierce Biotechnology, Rockford, IL, USA) according to the manufacturer's protocol.

### 2.5. Electrophoretic Mobility Shift Assay

NF*κ*B activity was evaluated by electrophoretic mobility shift assay (EMSA) 2 h after stimulation (*n* = 3/group). Nuclear extracts used in supershift and competition experiments were harvested from snap frozen lungs as described above. The NF*κ*B oligonucleotides (Santa Cruz Biotechnology Inc., Santa Cruz CA, USA) NF*κ*B: 5′-AGT TGA GGG GAC TTT CCC AGG C-3′ (sc-2505)) were end-labelled with (*γ*-32P) ATP (Amersham, Freiburg, Germany). 

Binding reactions (25 *μ*L total) were performed by incubating 20 *μ*g of nuclear extracts for 30 min at room temperature with 4 mM Tris-Cl (pH 7.9), 12 mM HEPES, 1 mM DTT, 60 mM KCl, 10% glycerol, 1 mM EDTA, 2 mg poly(dI-dC)-poly(dI-dC), and 20,000 cpm of the labelled NF*κ*B oligonucleotide. The specificity of the DNA-protein binding was determined by competition with a 50-fold molar excess of the unlabeled NF*κ*B. The DNA-protein complexes were electrophoresed for 2 h at 30 mA in a 4% polyacrylamide gel in 0.5% tris-borate-EDTA running buffer. The gels were dried for 1 h, exposed overnight to imaging plates, and scanned with a phosphoimager (FLA3000, Fuji, Düsseldorf, Germany).

### 2.6. Ribonuclease Protection Assay

Cytokine-, chemokine-, MMP-, and TIMP mRNA levels (*n* = 4–6/group) were analysed with ribonuclease protection assay (RPA) 4 h after stimulation. For RPA, lungs were flash-frozen in liquid nitrogen and kept at −80°C. The tissue was homogenized and total RNA was extracted by guanidinium thiocyanate method as described elsewhere [23].

The mRNA levels of MMP-1, -2, -3, -8, -9, and TIMPs 1–4 per 20 *μ*g RNA sample were analysed with mMMP-1 multiprobe template set (BD Biosciences Pharmingen, San Diego, CA, USA). Chemokine mRNA of lymphotactin, RANTES (regulated on activation and expressed/secreted by T cells), macrophage inflammatory protein (MIP)-1*α*, MIP-1*β*, MIP-2, macrophage chemotactic peptide (MCP)-1, T-cell activation protein (TCA)-3, eotaxin, and interferon-inducible protein (IP)-10 was detected with mCK-5c multiprobe template set (BD Biosciences Pharmingen, San Diego, CA, USA). mRNA expression of proinflammatory cytokines IL-12p35, IL-12p40, TNF-*α*, IL-1*α*, IL-1*β*, IL-6, and IFN*γ* as well as receptor expression of TLR2 and TLR4 were determined with custom-made template sets (BD Biosciences, Heidelberg, Germany). Signals were quantified densitometrically with AIDA software v3.5 (Raytest, Straubenhardt, Germany) and normalized to ribosomal housekeeping gene L32.

### 2.7. Real Time – Quantitative PCR

TLR1, -6, and -9 gene expression (*n* = 6/group) was determined with RT-qPCR 4 h after stimulation. TNF-*α* mRNA was monitored with the same technique 0, 2, 4, and 6 h after TLR-ligand application. The TaqMan Gene Expression Assays (Applied Biosystems, Foster City CA, USA) for murine TLR1 (comm. : Mm00446095 m1), TLR6 (comm. : Mm02529782 s1), TLR9 (comm. : Mm00446193 m1), TNF-*α* (comm. : Mm00443258 m1), and murine GAPDH (comm. : Mm999999915 g1) as housekeeping gene were used. RT-PCR was performed according to the manufacturer's protocol.

### 2.8. Enzyme Linked Immunosorbent Assay

TNF-*α* protein expression (*n* = 4/group) was determined with enzyme linked immunosorbent assay (ELISA; BD Biosciences, San Jose, CA, USA) 0, 2, 4, and 6 h after stimulation. For protein isolation, pulmonary tissue was homogenized and incubated on ice for 5 min in 1 mL ELISA buffer containing protease inhibitors (Roche, complete mini no. 11836153), PBS, Triton X-100 (1 *μ*L/mL, Sigma Aldrich, Munich, Germany), phenylmethylsulfonyl-fluoride (PMSF, 250 mM in isopropanol, 1 *μ*L/mL, Roche, Basel, Switzerland). Samples were incubated on ice for 20 min, homogenized, and centrifuged 15 min at 4°C and 13,110 × g. The supernatant was used for measuring intrapulmonary TNF-*α* protein levels in a microplate reader (Expert 96, Asys Hitech, Eugendorf, Austria).

### 2.9. Zymographic Activity Assay

For zymographic activity of pro and active forms of MMP-2 and -9 in lung tissue (*n* = 3/group), 60 *μ*g protein were mixed with 2x tris-glycine SDS sample buffer, and loaded on 10% polyacrylamide gels (SDS-PAGE) copolymerized with gelatin (each 0.3 mg/mL type A from porcine skin and type B from bovine skin; Sigma Aldrich, Munich, Germany). Following 90 min of electrophoresis at 125 V, gels were washed with 2.5% Triton X-100 (Sigma Aldrich) for 3 × 20 min to remove SDS. Afterwards, gels were incubated for 48 h at 37°C in developing buffer (50 mM Tris HCl, 0.2 M NaCl, 5 mM CaCl_2_, 0.02% Brij). Gels were stained using 0.1% (w/v) Brilliant Blue (Sigma Aldrich) in a mixture of water : methanol : acetic acid (5 : 5 : 1 v/v), destained in 45% methanol, and 3% acetic acid in water (v/v). Areas of protease activity were detected as transparent bands against blue background. All gelatinolytic activities reported could be inhibited by addition of EDTA to incubation buffer. Zymograms were scanned, and signals were quantified using AIDA software (AIDA Image Analyser, Raytest GmbH, Straubenhardt, Germany). For comparison, the zymographic activity of the three virulence factors 4 h after stimulation was normalized to their baseline activity (0 h, data not shown).

### 2.10. Determination of Myeloperoxidase Activity in Pulmonary Tissue

After 4 h of TLR ligand stimulation, lungs were taken and flash frozen in liquid nitrogen (*n* = 5/group). Tissue was homogenised on ice in 1 mL of 0.5% hexadecyltrimethyl ammonium bromide (HTAB; H-5882, Sigma) in 50 mM potassium phosphate buffer (1 mL buffer/50 mg tissue). 1 mL of the homogenate was transferred into a tube and centrifuged at 5,000 rpm for 4 min. 7 *μ*L of the supernatant were mixed with 200 *μ*L o-dianisidine solution (16,7 mg o-dianisidine (D-3252, Sigma) in 90 mL ddH_2_O, 10 mL 50 mM potassium phosphate buffer, and 50 *μ*L 1% H_2_O_2_). The change in absorbance with time was continuously recorded at 450 nm using a kinetic microplate reader.

### 2.11. Statistics

All values are expressed as means ± SEM. Significant differences among experimental groups of *P* < .05 are indicated. One-way ANOVA was used to determine significant differences in mRNA-expression (*n* = 4), ELISA (*n* = 4), and zymographic assay (*n* = 3) between the different stimulation groups. When appropriate, Bonferroni post hoc testing was performed. Statistics were calculated using Prism 5.0 (GraphPad Software Inc., San Diego, CA, USA).

## 3. Results

### 3.1. TNF Activity

To equilibrate the potency of the different applied TLR ligands a TNF activity assay was applied. Viability of TNF-sensitive fibroblasts exposed to serum from animals treated by one of the three TLR ligands dropped significantly in all groups (LTA: 57 ± 13.7%, LPS: 40 ± 2.4%, CpG-ODN: 42 ± 5.1%; *P* < .05 versus PBS serum control) 2 h after incubation. This indicated a strong inflammatory reaction to all three pathogens. There was no statistical difference in viability between the stimulated groups. We concluded that the applied virulence factors exhibit equipotency concerning TNF induction.

### 3.2. NF*κ*B Activity

**Figure 1:**
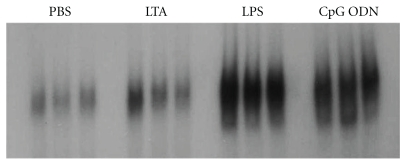
NF*κ*B activation. Representative EMSA for pulmonary nuclear NF*κ*B activity in LTA-, LPS- and CpG-ODN-stimulated mice (*n* = 3/group) after 2 h. Each lane represents an individual animal. LPS induced the strongest signal followed by CpG-ODN and LTA.

NF*κ*B is an essential transcription factor of inflammatory signalling. Hence, NF*κ*B transcriptional activation was measured 2 h after stimulation with the three TLR ligands using an EMSA. All virulence factors increased the activity of the transcription factor compared to baseline. No major visual differences were revealed following LPS and CpG-ODN stimulation (Figure 1). LTA, however, induced a markedly lower NF*κ*B activity than the other two ligands. 

### 3.3. TLR mRNA Expression

**Figure 2:**
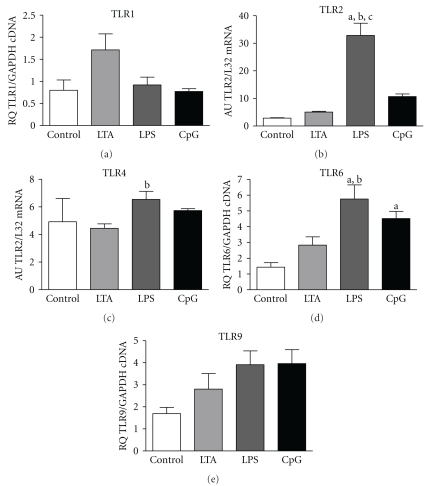
TLR mRNA expression. Analysis of TLR1, 2, 4, 6, and 9 in control, LTA-, LPS-, and CpG-ODN-stimulated pulmonary tissue (TLR1/6/9 detected with RT-qPCR, values depicted as relative quotient (RQ) to housekeeping gene; TLR2/4 detected with RPA, values expressed as arbitrary units (AU) normalized to L32 mRNA,). Alphabetic characters indicate *P*-values < .05 from Bonferroni post hoc testing (a = versus control, b = versus LTA, c = versus CpG-ODN; M ± SEM; *n* = 6/group).

Sensitivity of pulmonary tissue to LPS, CpG-ODN and LTA may be regulated by the level of specific TLR expression. Therefore, we monitored mRNA expression of TLR1, 2, 4, 6, and 9 4 h after stimulation. LTA application resulted in a twofold increase of TLR1, 2, and 6 expression but did not reach the level of significance (Figure 2), neither TLR4 nor TLR9 mRNA was changed. LPS acted more strongly elevating TLR2 and 6 expressions significantly versus control and TLR4 versus LTA-treated animals. CpG-ODN raised TLR6 mRNA exclusively.

### 3.4. Time Course of TNF-*α* mRNA and Protein Expression

TNF-*α* has been shown to be an early inflammatory marker, which is often upregulated transiently [24]. Therefore, we monitored the time course of pulmonary TNF-*α* mRNA expression 0, 2, 4, and 6 h after virulence factor challenge (Table 1, *n* = 4/group). TNF-*α* mRNA was significantly induced 2 h after LTA and CpG-ODN. LPS challenge, however, raised mRNA expression significantly at all investigated time points. Under baseline conditions, TNF-*α* protein expression was not detectable in ELISA. 2 h after stimulation, all virulence factors significantly increased TNF-*α* protein expression (Table 1). At the later time points, a significant enhancement of TNF-*α* protein was observed in the LPS group only. 

### 3.5. Cytokine and Chemokine mRNA Expression

**Figure 3:**
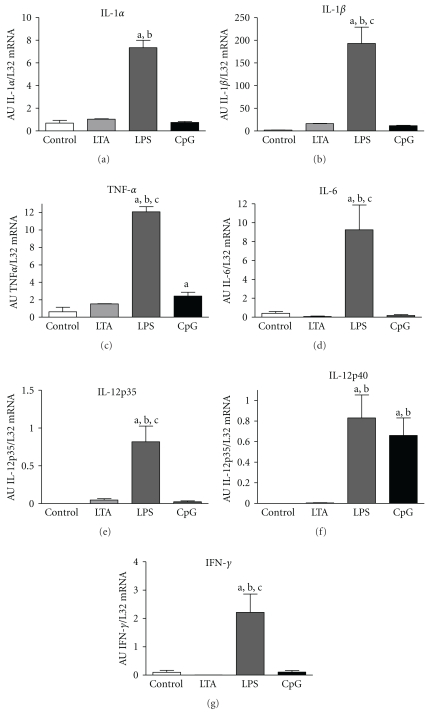
Cytokine mRNA expression. Densitometric analysis of IL-1*α*, IL-1*β*, TNF-*α*, IL-6, IL-12p35, IL-12p40, and IFN-*γ* in control, LTA-, LPS-, and CpG-ODN-stimulated pulmonary tissue. Values of mRNA expressions are normalized to L32 and expressed as arbitrary units (AU). Alphabetic characters indicate *P*-values < .05 from Bonferroni post hoc testing (a = versus control, b = versus LTA, c = versus CpG-ODN; *n* = 4; M ± SEM; *n* = 6/group).

The amount of cytokine and chemokine mRNA expression reflects the severity of inflammation. Therefore, these mediators were investigated in the lung 4 h after stimulation using RPA (Figure 3). LTA stimulation did not significantly induce any cytokine mRNA expression. In contrast, LPS stimulation induced a significant increase of all tested cytokines IL-1*α*, IL-1*β*, TNF-*α*, IL-6, IL-12p35, IL-12p40, and IFN-*γ*, whereas CpG-ODN only elevated TNF-*α* and IL-12p40 mRNA. 

**Figure 4:**
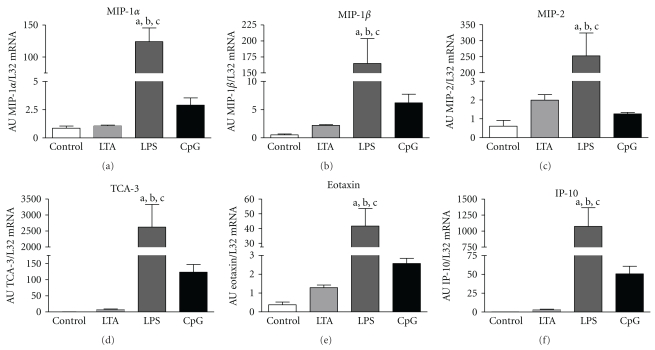
Chemokine mRNA expression. Densitometric analysis of MIP-1*α*, MIP-1*β*, MIP-2, TCA-3, eotaxin, and IP-10 in control, LTA-, LPS-, and CpG-ODN-stimulated pulmonary tissue. Values of mRNA expressions are normalized to L32 and expressed as arbitrary units (AU). Alphabetic characters indicate *P*-values < .05 from Bonferroni post-hoc testing (a = versus control, b = versus LTA, c = versus CpG-ODN; *n* = 4, M ± SEM; *n* = 6/group).

Analysis of lungs from LPS-treated mice revealed a marked and significant increase in all monitored chemokines (lymphotactin, RANTES, MCP-1, MIP-1*α*, MIP-1*β*, MIP-2, TCA-3, eotaxin and IP-10, Figure 4) as compared to all other groups. However, neither LTA nor CpG-ODN stimulation caused any significant changes in chemokines.

### 3.6. Myeloperoxidase Activity in Pulmonary Tissue

**Figure 5:**
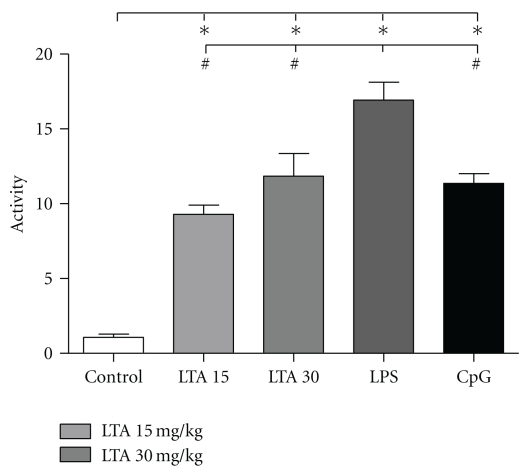
Myeloperoxidase activity in pulmonary tissue. Level of myeloperoxidase activity (x-fold of control) in lung tissue from control, LTA-, LPS- or CpG-treated mice 4 h after stimulation (*P < .01 versus control, ^#^ *P* < .01 versus LPS; M ± SEM; *n* = 5/group).

Myelop-eroxidase (MPO) activity was assayed as a marker of neutrophil infiltration into the lung. MPO activity was increased 9- to 16-fold in all stimulated groups 4 h after TLR ligand application (Figure 5). With a 16-fold induction compared to the control group, MPO activation was highest in lung tissue from LPS-treated mice. This value differed significantly from LTA- and CpG-stimulated mice which showed a 9- and 11-fold increase. Interestingly, doubling of the standard LTA dose did not further raise MPO activity.

### 3.7. MMP and TIMP mRNA Expression

**Figure 6:**
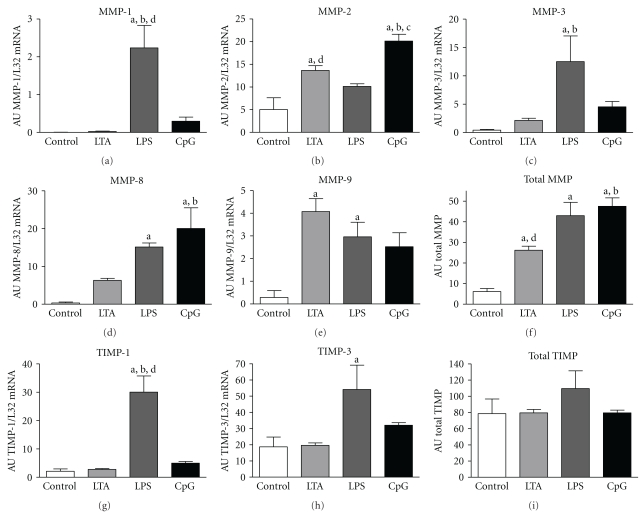
MMP, TIMP mRNA expression. Densitometric analysis of MMP-1, -2, -3, -8, 9, TIMP-1, and -3 in control, LTA-, LPS-, and CpG-ODN-stimulated pulmonary tissue as well as total MMP and TIMP expression. Values of mRNA expressions are normalized to L32 and expressed as arbitrary units (AU). Alphabetic characters indicate *P*-values < .05 from Bonferroni post-hoc testing (a = versus control, b = versus LTA, c = versus LPS, d = versus CpG-ODN; M ± SEM; *n* = 4/group).

During inflammation extracellular matrix remodelling may occur. The presence of MMPs and TIMPs indicates current modification of lung tissue. Consequently, 4 h after simulation total MMP mRNA expression was significantly enhanced by all three virulence factors with the greatest overall stimulus being CpG-ODN (Figure 6). LTA induced a significant increase in MMP-2 and MMP-9 as well as a 5-fold elevation in MMP-3 and 18-fold elevation in MMP-8. LPS stimulation significantly enhanced MMP-1, MMP-3, MMP-8, and MMP-9 while CpG-ODN raised MMP-2 and MMP-8 significantly as well as MMP-1, MMP-3, and MMP-9 nonsignificantly. 

Furthermore, LPS significantly enhanced TIMP-1 and -3 expressions whereas LTA and CpG-ODN did not induce any changes in single TIMP expressions. Interestingly, the unchanged TIMP-2 expression was relatively high in all groups (control: 57.78 ± 14.79; LTA: 57.08 ± 2.63; LPS: 25.38 ± 1.68; CpG-ODN: 42.56 ± 2.80) compared to TIMP-1 and -3. 

Since the stoichiometry between the gene expression of MMPs and their physiological antagonist (TIMPs) has been shown to be important for the regulation of the extracellular matrix remodelling, we calculated the ratios of MMPs to TIMPs (Table 2). Due to the up-regulation of the total MMP mRNA expression in all stimulated groups, overall MMP/TIMP ratio was significantly elevated 4-fold by LTA, 5-fold by LPS, and 8-fold by CpG-ODN.

### 3.8. Zymographic Activity of MMP-2 and MMP-9

**Figure 7:**
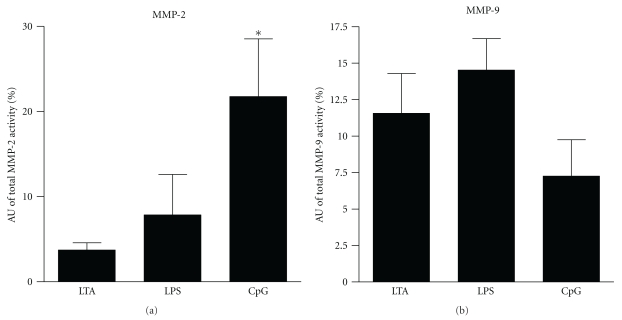
MMP zymographic assay. Percentage of total quantified MMP-2 or MMP-9 activity, respectively. Zymographic activity after stimulation (4 h) was normalized to baseline activity (0 h). Values are expressed as arbitrary densitometric units (AU; *P < .05 versus LTA and LPS; *n* = 3/group).

To expand the results on MMP and TIMP mRNA expression, the activity of MMP-2 and MMP-9 was detected in a zymographic assay. Accordingly, we detected an induction of MMP-2 and MMP-9 in their precursory (pro) and active form (not shown). To compare effects of different virulence factors, we normalized zymographic activity (4 h) to baseline activity (0 h) (Figure 7). CpG-ODN challenge significantly increased MMP-2 activity compared to LPS and LTA application. Here, up-regulation was mainly due to 72 kDa proMMP-2 activity. MMP-9 activity was enhanced comparably by all stimuli.

## 4. Discussion

With the present study, we characterized differential pulmonary inflammatory responses to various virulence factors. Studies comparing the influence of different TLR stimuli on pulmonary tissue were mainly performed *in vitro* [25]. Therefore, we decided to compare LTA, LPS, and CpG-ODN challenge using remote stimulation *in vivo* to simulate the initiation of sepsis. For characterization of the inflammatory cascade we monitored a variety of endpoints like TLR expression, NF*κ*B activation, expression of various cytokines and chemokines, and MPO activity as well as expression of MMP/TIMPs. In the lung, this broad combination of TLR stimuli and endpoints detected *in vivo* has not yet been performed. 

To provide comparability of results, we defined equipotent concentrations for LTA, LPS, and CpG-ODN via a TNF activity assay. TNF induction as a measure for equipotency of inflammatory stimuli has been established earlier [25]. After stimulation with LPS we observed a strong proinflammatory reaction with increased NF*κ*B activation, enhanced cytokine and chemokine expression, and a differential response in MMP and TIMP expression. The equipotent dose of CpG-ODN initiated mediator expression to a lesser degree. Here, cytokine and chemokine expression remained a multiple below values evoked by LPS. Interestingly, stimulation with an equipotent dose of LTA had a markedly lesser impact on the induction of cytokines and chemokines in lung tissue. Significant variations only occurred in TNF-*α* expression, MPO activity, and MMP/TIMP regulation. 

In order to understand the differential answers to TLR stimulation better, we investigated the expression of TLR1, 2, 4, 6, and 9. LPS induced an increased expression of TLR2, 4, and 6 whereas CpG-ODN elevated solely the TLR6 expression. LTA did not change any investigated TLR. Induction of TLR2 and 4 by LPS in pulmonary tissue has already been observed by others [26]. Interestingly, pulmonary TLR4 mRNA expression did not reveal a strong regulation after LPS challenge. In contrast, TLR2 expression was very sensitive to LPS, as it was increased more than 10-fold. In this context, it is interesting that also TLR6 is upregulated by LPS and CpG-ODN challenge, as LTA signalling depends on heterodimerization of TLR2 and TLR6 or TLR2 and TLR1, and CD36 [27]. This up-regulation of TLR2 and TLR6 might sensitize the lung in an ongoing inflammation to gram-positive stimuli. In accordance with our results, it has been shown that bronchial epithelium regulates its sensitivity to recognize microbes by managing TLR expression levels [27]. Bronchial epithelial cells could be stimulated *in vitro* only marginally by gram-positive bacteria bearing known TLR2 ligands or with LTA alone while gram-negative bacteria were easily recognized. As mucosal surfaces are prone to contact with pathogenic, as well as nonpathogenic microbes, immune recognition principles have to be tightly regulated to avoid uncontrolled permanent activation. Hence, airway epithelium displays a low sensitivity to inhaled gram-positive bacteria whereas gram-negative bacteria, which are rarely found in airways, do easily induce epithelial activation [27]. A strong immune response following a first hit with LPS and a moderate induction caused by first hit with LTA might be expected in the lung. However, due to the observed up-regulation of TLR2 by gram-negative challenge sensitivity to LTA may be elevated during a second hit. CpG-ODN-dependent-up-regulation of TLR6 detected here may further support the sensitization to LTA during ongoing inflammation. However, significant up-regulation of pulmonary TLR9 expression was not demonstrated here and is in accordance with recent results from our group [14]. 

Cytokine release evoked by TLR stimulation on lung tissue has been investigated in different settings, such as cultured pulmonal cells or by application of TLR ligands to the airway side [12, 14]. To our knowledge, this study compares for the first time a challenge with different TLR ligands applied systemically. TNF-*α* taken from the plasma was used to determine equipotent doses of remote stimuli. On the other hand, TNF-*α* mRNA and protein were investigated in pulmonary tissue to compare the inflammatory response of the lung from 0–6 h after stimulation with each TLR-ligand. Here, LPS led to a significant increase of TNF-*α* mRNA and protein at all time points while LTA and CpG-ODN elevated its expression only at the earliest time point (2 h). These results confirm the well-known observation that TNF-*α* is an early inflammatory marker [24]. Apart from TNF-*α*, LPS also induced all other investigated proinflammatory cytokines at 4 h, CpG-ODN raised only IL12p40 while LTA failed to elevate any of the other inflammatory markers. These findings further support the above-mentioned low sensitivity of the lung to gram-positive stimuli, which may be overcome by direct intrapulmonary application [28, 29]. We found a significant up-regulation of IL-12p40 *in vivo* in pulmonary tissue by CpG-ODN and LPS. This transfers the results from Albrecht et al. [25] to the* in vivo *situation, as they demonstrated an induction of IL-12p40 mainly by CpG-ODN in transfected macrophages and dendritic cells. IL-12p40 bridges the gap between innate and adaptive immunity because it is potently regulated in antigen-presenting cells, thereby activating the adaptive T-helper (T_H_1) cells. 

These immune cells are attracted into the site of inflammation by chemokines [30]. In our study, significant chemokine induction was only found after LPS stimulation. This is in accordance with the observed high MPO activity after TLR4 stimulation, as monocytes and neutrophils attracted by chemokines exclusively release MPO. LPS-dependent infiltration of polymorphonuclear neutrophils into pulmonary vasculature, lung interstitium, and alveolar space as well as bronchoalveolar lavage fluid has already been demonstrated by others [31]. In our setting of remote stimulation, CpG-ODN, and LTA failed to raise chemokine expression. At the first glimpse, this seems to be in contrast to the literature [28, 32, 33]. However, this difference between our results and those of others may be attributed to the diverse mode of administration (local versus systemical). 

MMPs play a decisive role in the repair of the alveolar epithelium during pulmonary inflammation by cleaving components of the extracellular matrix (ECM) [34]. However, excessive expression of MMPs can also destroy the ECM and initiate further inflammation and changes in pulmonary architecture. MMP-2 and -9 can be produced by various resident cells in the lung [35] or might stem from stimulated immune cells. Similar to MMPs, the defined role of their inhibitors is also not fully understood in sepsis. Some studies indicate that high TIMP-1 levels are associated with severe forms of the disease and might, therefore, serve as possible prognostic markers of poor survival [36]. Despite low mRNA levels of cytokines and chemokines in the LTA group, MMP-2 and -9 were significantly induced in lung tissue as a sign of inflammatory affection. However, TIMP-1 expression was low and remained unaffected by LTA. A high MMP9/TIMP1 ratio has been shown to be associated with a less severe progression of different lung diseases [37–39]. LPS challenge significantly elevated TIMP-1 and -3. As mentioned above, high TIMP-1 expression is associated with severe lung disease. TIMP-3 may act in a similar manner as TIMP-3-deficient mice were protected from sepsis-induced pulmonary inflammation and remodelling [40]. 

The present study provides detailed information on inflammatory events in the lung after *in vivo* challenge with equipotent doses of LTA, LPS, and CpG-ODN in a murine model of sepsis. These different remote stimuli resulted in specific inflammatory responses in pulmonary tissue indicating an organ-specific immune modulation.

LTA induced the lowest number of inflammatory mediators, which was associated with a mild progression of lung remodelling. As gram-positive bacteria are commonly inhaled, the respective immune response of the lung has to be strictly controlled to avoid autodestructive inflammation. On the other hand, gram-negative bacteria enter the respiratory tract much more rarely, which may explain the stronger inflammatory response to LPS in our setting. However, an ongoing infection with gram-negative bacteria might sensitize the lung towards gram-positive stimuli as TLR2 and TLR6 expressions are upregulated by LPS. In addition, CpG-ODN that induces the inflammatory cascade via TLR9 is released by both gram-positive and gram-negative bacteria. Thus, in case of a bacterial sepsis, the inflammatory response in the lung will never be induced by exclusive activation of TLR2 or TLR4 but always by costimulation of TLR9.

Our findings indicate that the lung may be more susceptible to systemic inflammation caused by gram-negative stimuli than to one elicited by gram-positive stimulation.

##  Conflict of Interests

The authors have no conflict of interests.

## Figures and Tables

**Table 1:** TNF-*α* expression in the lung. Pulmonary TNF-*α* mRNA (RQ TNF-a/GAPDH cDNA) and TNF-*α* protein (pg/mg protein) expression 0, 2, 4, and 6 h after LTA-, LPS-, and CpG-ODN-stimulation. Significant differences to control values are indicated (*n* = 4; *P < .05; bdl = *below detection limit*; M ± SEM; *n* = 6/group).

	TNF-*α* mRNA (RQ TNF-*α*/GAPDH cDNA)	TNF-*α* protein (pg/mg protein)
0 h LTA	1.11 ± 0.11	0.00 ± 0.00 (bdl)
2 h LTA	5.79 ± 0.68*	16.05 ± 7.89*
4 h LTA	2.08 ± 0.36	0.00 ± 0.00 (bdl)
6 h LTA	1.72 ± 0.37	0.00 ± 0.00 (bdl)

0 h LPS	0.61 ± 0.07	0.00 ± 0.00 (bdl)
2 h LPS	14.31 ± 1.36*	24.36 ± 3.63*
4 h LPS	12.82 ± 2.05*	12.35 ± 2.74*
6 h LPS	12.70 ± 1.05*	9.78 ± 0.42*

0 h CpG	0.54 ± 0.02	0.00 ± 0.00 (bdl)
2 h CpG	19.88 ± 3.90*	12.81 ± 1.55*
4 h CpG	2.65 ± 0.67	2.70 ± 0.84
6 h CpG	2.74 ± 0.71	3.84 ± 2.92

**Table 2:** MMP/TIMP mRNA ratios. MMP/TIMP mRNA ratios in control, LTA-, LPS- and CpG-stimulated pulmonary tissue. Values of mRNA expressions are normalized to L32 and expressed as arbitrary units (AU). Alphabetic characters indicate *P*-values < .05 from Bonferroni post-hoc testing (a = versus control, b = versus LTA, c = versus LPS, d = versus CpG-ODN; M ± SEM; *n* = 4/group).

	Control	LTA		LPS		CpG	
MMP-l/TIMP-l	0.00 ± 0.00	0.00 ± 0.00	c, d	0.07 ± 0.01	a, b, d	0.06 ± 0.02	b
MMP-2/TIMP-l	2.29 ± 0.13	5.10 ± 0.79	a, c	0.38 ± 0.08	b, d	4.19 ± 0.43	c
MMP-3/TIMP-l	0.22 ± 0.05	0.81 ± 0.20		0.39 ± 0.07		0.95 ± 0.44	a
MMP-8/TIMP-l	0.15 ± 0.02	2.28 ± 0.15		0.55 ± 0.09	d	4.45 ± 1.73	a, c
MMP-9/TIMP-l	0.11 ± 0.04	1.44 ± 0.08	a, c, d	0.12 ± 0.05	b	0.55 ± 0.18	b
MMP-l/TIMP-2	0.00 ± 0.00	0.00 ± 0.00	c	0.09 ± 0.02	a, b, d	0.01 ± 0.00	c
MMP-2/TIMP-2	0.09 ± 0.01	0.24 ± 0.16	d	0.41 ± 0.04	a, b, d	0.48 ± 0.05	a, b, c
MMP-3/TIMP-2	0.00 ± 0.00	0.04 ± 0.01	c	0.48 ± 0.15	a, b, d	0.11 ± 0.02	C
MMP-8/TIMP-2	0.01 ± 0.00	0.11 ± 0.01	c, d	0.60 ± 0.05	a, b	0.48 ± 0.13	a, b
MMP-9/TIMP-2	0.00 ± 0.00	0.07 ± 0.01		0.12 ± 0.04	a	0.06 ± 0.02	
MMP-l/TIMP-3	0.00 ± 0.00	0.00 ± 0.00	c	0.04 ± 0.01	a, b, d	0.01 ± 0.00	c
MMP-2/TIMP-3	0.27 ± 0.04	0.70 ± 0.06	a, c	0.26 ± 0.10	b, d	0.64 ± 0.07	a, c
MMP-3/TIMP-3	0.03 ± 0.01	0.11 ± 0.02		0.24 ± 0.05	a	0.14 ± 0.03	
MMP-8/TIMP-3	0.02 ± 0.00	0.32 ± 0.00		0.37 ± 0.12		0.61 ± 0.15	a
MMP-9/TIMP-3	0.01 ± 0.01	0.21 ± 0.02	a, d	0.09 ± 0.05		0.08 ± 0.02	b

Total MMP/TIMP	0.08 ± 0.01	0.33 ± 0.01	a, d	0.42 ± 0.08	a	0.60 ± 0.05	a, b
